# Tool development to measure the resilience of water supply systems in Tanzania: Economic dimension

**DOI:** 10.4102/jamba.v13i1.860

**Published:** 2021-01-11

**Authors:** Lukuba N. Sweya, Suzanne Wilkinson

**Affiliations:** 1 Department of Civil and Environmental Engineering, Faculty of Engineering, The University of Auckland, Auckland, New Zealand; 2 School of Built Environment, Massey University, Albany Campus, Auckland, New Zealand

**Keywords:** resilience, floods, water supply systems, Delphi techniques, economic dimension

## Abstract

The world has experienced devastating disasters causing severe human life and economic losses, which is estimated to be 68.5% of the global economic losses between 2005 and 2017. Natural disasters are of great concern – they caused total damage of approximately $3.5 trillion during the past century – which is more than the global infrastructure development investment in 2014. Floods – exacerbated by climate change – are expected to cause more damages, and water supply infrastructures will continue to suffer if resilience is not improved. Measuring the economic changes affecting resilience would assist in developing risk reduction initiatives to minimise disaster losses. Such a measure is lacking for Tanzania water supply systems (WSSs). The current article applied three-stage processes – literature review, pre-assessment and Delphi technique – to develop a resilience tool to measure economic resilience for urban WSSs in Tanzania. Thematic and standard descriptive analyses were carried out during the study. Dynamism principle and three indicators – system investment proportionality, public–private partnership and cost recovery – emerged as principal components for the tool. The tool is expected to be useful during water authorities’ planning processes and budgeting in order to improve the overall WSSs resilience.

## Introduction

Human civilisation has become a superorganism, changing the environment from which it evolved inducing new hazards with no analogue – with increasing complexity and interaction of human, economic and political systems within ecological systems, the risk becomes increasingly systemic (UNDRR 2019). The civilisation’s contribution to the already changing environment is expected to yield more frightening impacts on the economy and human lives if no serious interventions are taken. The ability to exacerbate the impacts of environmental-related disasters is particularly a concern – from the 1960s, to date, there have been an exponential increase in natural disasters’ frequency, magnitude and impacts in terms of human life and economic losses (EM-DAT 2019). Globally, there are already significant losses – approximately $3.5 trillion – caused by natural disasters since 1900 (EM-DAT 2019), which is estimated to be more than the global infrastructure development investment in 2014 – approximately $3.4 tn (Bhattacharya et al. 2016). US$5.2 billion and approximately $136.8bn were spent on disasters risk reduction and response between 2005 and 2017 (UNDRR 2019). About 68.5% of all global economic losses from 2005 to 2017 were attributed to extensive risk events and heavily absorbed by the low-income households and communities, particularly in the low- and middle-income countries in Asia, Pacific and Africa (UNDRR 2019). Also, disasters’ reporting data are imperfect – particularly in developing countries – thus, disaster losses remain significantly unreported compromising accurate calculations of impacts and affecting the preparedness and mitigation plans for future events (UNDRR 2019).

In the case of Tanzania, only 5.7% of the internationally reported disasters had information, which represented the total damage for the past century (EM-DAT 2019). The country was the first in the top 10 countries for disaster damages in 2016 in terms of the percentage Gross Domestic Product (Guha-Sapir et al. 2017). Also, the country loses approximately $2bn annually because of flooding hazard – two times the state budget for the ministry of health, education, home affairs and environment combined for the financial year 2017–2018 (https://www.dailynews.co.tz/news/floods-cost-tanzania-us-2billion-annually.aspx). During the disasters, infrastructures are not immune; the risk of coastal flooding is projected to have more significant damage than that of riverine floods, and thus, damages to infrastructure and assets are increasing (UNDRR 2019), needing investment in resilience building to

- avoid losses when disasters strike,
- stimulate economic activities from reduced risks, and
- develop co-benefit or uses of a specific disaster risk management investment (UNDP 2019).

Water supply systems (WSSs) encompass catchments/water sources, water treatment facilities, distribution networks, raw water and clean water transmission pipes, electronic facilities and cyber systems (Van Leuven 2011). The Tanzania urban WSSs encompass 26 Regional Water Supply and Sanitation Authorities (R-WSSAs) and eight National Project Water Supply and Sanitation Authorities (NP-WSSAs) in the Mainland-Tanzania. These authorities are the principle water service providers, contributing to an average of 74.2% and 55%, respectively, of the population in their regions (URT 2018). National Project Water Supply and Sanitation Authorities, such as Kahama Shinyanga Water Supply and Sewerage Authority (KASHUWASA), are also bulk water suppliers to other WSSAs. Others are privately owned infrastructures, community-based organisation facilities, individual boreholes and direct fetching of water from streams, rivers, lakes and locally dug wells. The WSSAs are the lawful organisations providing public water services in the urban areas (*Water Supply and Sanitation Act* 2019). Such organisations – in collaboration with the Ministry of Water – are responsible for developing and implementing mitigation measures to minimise disaster impacts on WSSs (*Disaster Management Act* 2015). The WSSAs’ main source of income is through billing; however, the majority operate under license III – still get financial, managerial and technical support from the government and partially cover their operational costs (URT 2020) – thus, are unable to collect enough funds that can enhance contingency planning to prepare for disaster impacts.

The possibility to measure the economic changes triggered by disasters is a crucial step towards disaster risk reduction (Renschler et al. 2010). The economic dimension includes economic factors driving the restoration process of urban infrastructures and recovery processes – before, during and after natural disasters – needing to be determined to select optimal resources allocation and preparedness measures right after an extreme event (Martinelli et al. 2014). Researchers have shown the importance of economic factors and their measurements for the performance of infrastructures worldwide (Balaei et al. 2018; Bhattacharya et al. 2016; Bruneau et al. 2003; De Bruijn et al. 2017; Vugrin et al. 2010) during disasters. However, the current global resilience gap is lacking the universal measurement tool for all systems, more so for economic patterns and their consequences differing in various systems. As such, there is a quest for localised approaches that would precisely measure the economic factors enhancing the resilience of WSSs – which has not been performed for Tanzania. Therefore, the current study applied Delphi techniques to develop a resilience tool to floods encompassing economic components that are useful in the urban WSSAs’ planning processes and budgeting in order to improve the resilience.

## Economic resilience

Resilience is a multidimensional concept that is being applied in various fields, such as ecology, social sciences, engineering and economics, to describe how the systems are better prepared to withstand, respond, recover and adapt to disaster impacts. Originally, according to Holling (1973), several studies have tried to define the concept without consensus on a universal definition (Vugrin et al. 2010). Some researchers develop generalised definitions, whereas others develop context-specific definitions focusing on specific dimensions, such as social dimension, economic dimension and organisational dimension. In all cases, the resilience concept is defined based on some or all four phases of disaster management – mitigation, preparedness, response and recovery. A meta-definition of resilience is given by Stevenson et al. (2015):

[*T*]he ability to absorb the effects of a disruptive event, minimize adverse impacts, respond effectively post-event, maintain or recover functionality, and adapt in a way that allows for learning and thriving, while mitigating the adverse impacts of future events. (p. 7)

The definition was developed by drawing together common attributes from 120 literature-based definitions. This definition provides a good platform for understanding the general concept of resilience, as applied in the current study.

Economic resilience is a more complex concept because the long-term investment in rehabilitation is complicated and unique post-disaster task (Bastaminia, Rezaei & Dastoorpoor 2017). The review of few definitions shows some discrepancies on how economic resilience is defined:

- The analysis of economic success with respect to the processes involved in disaster management (Christopherson, Michie & Tyler 2010),
- The capacity of an institution or a system to maintain its functions during crises (Rose 2004),
- Reconfiguration of economy, adaptability and infrastructure, and sustain acceptable growth in production, employment and welfare in the long term (Martin 2011),
- The ability of an economy or a local community to absorb and adapt to the negative effects of economic shock and move towards pre-disaster equilibrium or stability (Bastaminia et al. 2017) and
- The inherent ability and adaptive response that enables individual business firms and entire regions to avoid maximum potential loss (Rose & Liao 2005).

To suit the current study, common attributes apply encompassing factors affecting the functionality and recovery process aftermath, and determining options for households, communities, firms, water supply authorities at the time of flooding and potentially related disasters.

Likewise, measuring resilience is complex as there is no universal approach (Willis & Loa 2015) – various methodologies have been developed to operationalise and reduce concept’s ambiguity (Sharifi 2016). As such, studies have examined economic resilience empirically or with the use of simulation studies (Cutter 2016; Rose & Krausmann 2013) undergoing evolutions from Tierney (1997) to Rose and Liao (2005) who applied a computable general equilibrium (CGE). Also, using evidence-based such as the Federal Emergency Management Agency (FEMA’s) estimation tool, and survey (Kajitani & Tatano 2009). Other studies have treated economic aspects as a dimension/component to community resilience (Cimellaro et al. 2016; Mayunga 2007) and infrastructure resilience (Balaei et al. 2018; Bruneau et al. 2003; Vugrin et al. 2010) where several indicators apply to examine the resilience.

In all cases, Rose (2007) indicated that economic resilience is divided into *static* resilience and *dynamic* resilience – the former refers to the efficient use of resources at a particular point and time, whilst the latter implies to the repair and reconstruction affecting the time path of the economy. In each case, resilience emanates from both internal motivation (*internal resilience*) and the stimulation of private or public policy decisions (*adaptive resilience*). Moreover, economic resilience takes place at three levels – individual household or firm (*microeconomic level*), sectors (*mesoeconomic level*) and general economy (*macroeconomic level*) (Rose 2007, 2017; Rose & Krausmann 2013). Besides, the dominant economic factors include the economic structure, efficient use of resources to prepare and mitigate disasters, and repair and reconstruction aftermath (Rose 2007, 2016; Sharifi 2016). The current study evaluates economic factors at the society and water organisation levels, thereby analysing their internal economic capacity and the interaction with external stakeholders. As such, the resilience for Tanzania urban WSSs to flood hazards relate directly to micro-economy and mesoeconomy, with common factors that could affect the economic resilience presented in Table 1. Such factors were fundamental during the development of the current tool.

**TABLE 1:** Common factors used in assessing the economic resilience.

Principles	Indicators	Authors
1. Structure (Sharifi 2016)	Employment rate and opportunities	Alshehri, Rezgui and Li (2015), Bastaminia et al. (2017), Cimellaro et al. (2016), Mayunga (2007) Sharifi (2016)
	Income (equality, multiple sources …), poverty	Alshehri et al. (2015), Bastaminia et al. (2017), Cimellaro et al. (2016), Cutter (2016), Mayunga (2007), Sharifi (2016)
	Age structure of the working population	Sharifi (2016)
	Qualification of working age population	Sharifi (2016)
	Individuals with high and multiple skills; literacy (education)	Cimellaro et al. (2016), Sharifi (2016)
2. Static/security (Bastaminia et al. 2017; Cimellaro et al. 2016; Rose 2007; Sharifi 2016)	Individual and community serving	Mayunga (2007), Sharifi (2016)
	Collective ownership of community resources	Sharifi (2016)
	Insurance (domestic and non-domestic) and social welfare	Alshehri et al. (2015), Sharifi (2016)
	Financial instruments (contingency funds, operating funds, capital funds, etc.)	Sharifi (2016)
	Stability of prices and incomes, property value	Mayunga (2007), Sharifi (2016)
3. Dynamism (Bastaminia et al. 2017; Cimellaro et al. 2016; Rose 2007; Sharifi 2016)	Inward investment	Mayunga (2007), Sharifi (2016)
	Connection with the regional economy	Sharifi (2016)
	Business cooperation (inter and intra)	Sharifi (2016)
	Openness to micro-enterprises and micro-finance services, entrepreneurialism	Sharifi (2016)
	Public–private partnership	Sharifi (2016)
	Locally owned business and employers	Sharifi (2016)

Note: Most factors were extracted from Sharifi (2016), whose study reviewed 36 tools for measuring community resilience.

## Methodology

The current study was conducted to identify potential elements of the tool that can be applied to assess the economic resilience against floods for Tanzania urban WSSs. The tool refers to a framework of potential principles and indicators suitable for measuring the economic resilience for the country’s WSSs. The development of the tool relied on an initial review of the literature, a pre-assessment exercise, and a three-round Delphi process. The experts took part voluntarily based on their understanding of the objectives of this research.

**FIGURE 1:**
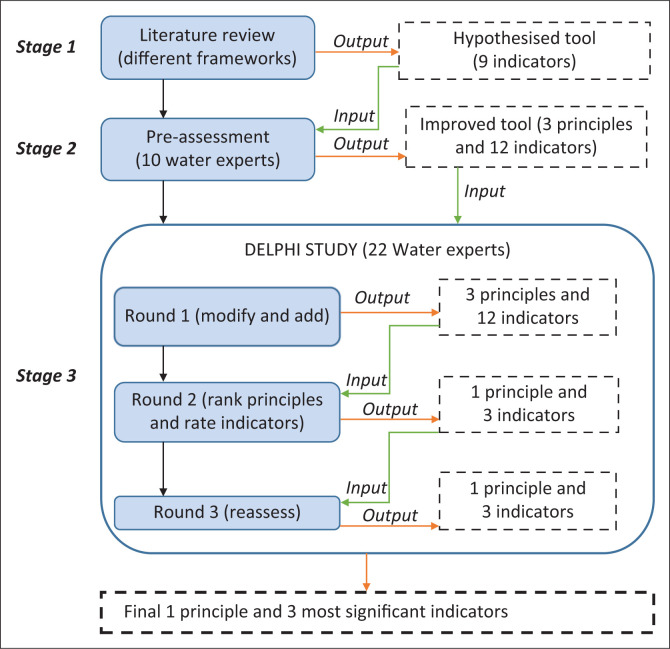
Processes used for developing the economic resilience tool.

The processes applied for developing the tool are presented in Figure 1. A literature review was conducted to identify key factors/indicators that had potential and could help inform the economic resilience for WSSs. The review was also reinforced with the water-related publications for Tanzania and international WSSs. The indicators selection relied on their adherence to one of the phases of disaster management – mitigation, preparedness, response and recovery. Also, the selection depended on the characteristics of resilience aligning with the water service-provision goals for the WSSs. Thus, a tool with nine indicators was proposed.

The proposed tool was made appropriate to assess resilience for Tanzania urban WSSs through a pre-assessment exercise taking place between September and October 2017, followed by a three-round Delphi survey from October 2018 to January 2019. A questionnaire was developed based on the hypothesised indicators and later used in the pre-assessment exercise. The pre-assessment involved 10 water supply experts from the public, private and research institutions. The experts had a working experience or research background of at least 5 years in the water supply industry. Experts were requested to comment, modify and add more indicators and rate their importance. The experts’ opinions improved the tool to three principles and 12 indicators.

A three-round Delphi study was introduced to further improve the tool and make it appropriate to Tanzania urban WSSs. The exercise included the 10 experts who had participated in the pre-assessment. Besides, 12 new experts were invited, making an overall panel of 22 experts. At the beginning of each round, all experts were physically contacted by the researcher and asked to develop consensus and prioritise the components of the tool. In the first round, participants were asked to comment on the principles and indicators, and add more indicators, whereas during the second and third rounds they were asked to rank the principles and rate the importance of the indicators. The ranking was determined based on the order of one for the most important principle to three for the least important principle. Rating relied on six key attributes – relevance, affordability, availability, reliability, simplicity and transparency (see Table 2) – as useful guidance for determining the indicators’ importance and potential for inclusion in the study.

**TABLE 2:** Key attributes for assessing economic resilience indicators.

Key attribute	Description	Reference
Relevance	The degree to which indicators are appropriate or related to this study	Balaei et al. (2018)
Affordability	Data accessible/generated for reasonable cost/level of effort	Morley (2012) from Villagran’s (2006)
Availability	Easy to collect and measure	Morley (2012) from Villagran’s (2006)
Reliability	Consistent over time	Morley (2012) from Villagran’s (2006)
Simplicity	Ease of understanding by decision makers	Cutter, 2014; Morley (2012) from Villagran’s (2006)
Transparency	Can the data be reproduced and verified?	Cutter 2014; Morley (2012) from Villagran’s (2006)

Each attribute was rated based on a five-point Likert scale: strongly disagree (1), disagree (2), neither agree nor disagree (3), agree (4) and strongly agree (5). The agreement was reached when at least 70% of experts, on average, had rated the indicators between 4 and 5 (Wakai et al. 2013), and the standard deviation of the ratings is between 0.3 and 0.998 (Zhong et al. 2015).

Thematic analysis was carried out manually for the comments obtained from the pre-assessment exercise and first-round Delphi survey. Standard descriptive statistical analysis was applied for second- and third-round data using Statistical Package for Social Sciences, IBM SPSS Statistics 25. The mean scores and median were used to establish the ranking order of the components of the tool, whereas mean scores and standard deviations were used to describe the group opinion and the convergence of the range of importance ratings of the indicators, respectively. Depending on whether the data were normally distributed and using *p* < 0.05 as the level of statistical significance, *t*-test or non-parametric Mann–Whitney test results were analysed to compare whether there was a significant difference between the second and the third round. Furthermore, Kappa statistics were calculated to show the percentage agreement between the two rounds.

### Ethical consideration

Ethical approval was obtained from the Ethics Committee of the University of Auckland, New Zealand (approval number 019619), and participant information sheets (PIS) were provided to experts and consent forms (CFs) signed by the participants.

## Results

The study was conducted to develop a tool that has components suitable for assessing the economic resilience for Tanzania WSSs. A three-stage approach was employed: literature review, pre-assessment and a three-round Delphi survey. A review of literature established key features leading to a hypothesised tool with nine indicators that are relevant in assessing the economic resilience for WSSs (see Table 3).

**TABLE 3:** Hypothesised indicators.

S/N	Indicator	Reference
1	Insurances for hazard events	Sharifi (2016)
2	Availability of funding for all elements of resilience planning including technical and organisational.	Sharifi (2016)
3	Qualification of the working age population	Sharifi (2016)
4	Openness to micro-enterprises and micro-finance services, entrepreneurism	Sharifi (2016)
5	Individuals with high and multiple skills; literacy (education)	Sharifi (2016)
6	Stability of prices and incomes, property value	Sharifi (2016)
7	Connections with regional economy	Fratesi and Senn (2009), Sharifi (2016)
8	Public–private partnership	Qian et al. (2020), Sharifi (2016)
9	Locally owned business and employers	Sharifi (2016)

### Pre-assessment

The proposed tool underwent a pre-assessment exercise involving 10 water experts – 50% possessed PhD qualifications, had more than 10-year experience in the water-related fields and were ranked as senior professionals at their workplaces. Others were senior associate professionals with experience ranging from 5 to 10 years. About 80% of all experts had experience in disaster management and possessed better research background. Results show that four indicators – ‘availability of funding for all elements of resilience’, ‘qualification of the working age population’, ‘individuals with high and multiple skills’ and ‘public, private partnership (PPP)’ – were accepted. Other indicators were rejected as shown in Table 4.

**TABLE 4:** Screening process for indicators during pre-assessment.

Assessment criteria	Number	Comments
1. At least 70% of experts agree or strongly agree 2. Standard deviation between 0.3 and 0.998	4	Accepted
Less than 70% of experts agree or strongly agree	3	Rejected
1. Less than 70% of experts agree or strongly agree 2. Standard deviation above 0.998	2	Rejected

Two indicators – ‘insurances for hazard events’, and ‘stability of prices and incomes’ were restored by researchers because the first influences the security of the affected population, infrastructures and organisations, whereas the second affects the ability of the people to obtain water services from alternative sources. Along with insurances, there was a need for assessing the savings behaviour of the people and the organisations’ cost recovery through billing, helping during crises. Thus, indicators such as ‘individual and community savings’ and ‘cost recovery’ were added. In addition to PPP, ‘business cooperation (intra and inter)’ indicator was added to assess the business-oriented interaction within and outside the organisations running the WSSs.

It is not uncommon to see high investment in parts of the WSSs than in others – especially in developing countries such as Tanzania. For instance, by 2015, the production part of the Dar es Salaam water supply had expanded twice the previous capacity, whilst the distribution part remained the same. This situation has resulted in pipe bursting and high levels of Non-Revenue Water (NRW) as the distribution system is unable to handle the water pressure. As such, researchers proposed an additional indicator – system investment proportionality. Moreover, through understanding the economic implications to the community, researchers proposed another indicator – expenditure on water services – to assess the water services expenses before and during flooding. Also, an ‘inward investment’ indicator was added to assess the stakeholders’ interests to invest in the water supply industry. The improved tool comprised 12 indicators – all indicators were grouped into the most dominant principles for assessing economic resilience: structure, security and dynamism (see Table 5).

**TABLE 5:** Improved tool from the pre-assessment exercise.

Principles	Indicators	Code	Reference
Structure	1. Employment rate and opportunities	EI1	Sharifi (2016)
	2. Income	EI2	Sharifi (2016)
	3. Expenditure on water services	EI3	†
Security	4. Individual and community savings	EI4	Sharifi (2016)
	5. Insurance for hazard events	EI5	Sharifi (2016)
	6. Stability of prices and incomes	EI6	Sharifi (2016)
Dynamism	7. Inward investment	EI7	Sharifi (2016)
	8. Business cooperation (intra and inter)	EI8	Sharifi (2016)
	9. Public-private partnership	EI9	Sharifi (2016)
	10. Funding	EI10	Hughes and Healy (2014)
	11. Cost recovery	EI11	†
	12. System investment proportionality	EI12	†

† , Indicators added by researchers based on experts’ opinions.

### Three-round Delphi survey

Amongst 22 experts who were initially contacted to participate in the exercise, 16 completed the first round, amongst those, 12 completed the second round and the third round. The response rates in the three rounds were 72.7%, 75% and 100%, respectively. No new expert was invited to participate after the exercise had commenced. The qualifications of the experts are presented in Table 6.

**TABLE 6:** Qualification of the experts involved in the three-round Delphi study for developing economic resilience tool.

Items	Categories	First		Second		Third	
		*N*	%	*N*	%	*N*	%
Education background	PhD	5	31.25	3	25.00	3	25.00
	Master	8	50.00	7	58.33	7	58.33
	Bachelor	3	18.75	2	16.67	2	16.67
Professional Rank	Senior professional	8	50.00	6	50.00	6	50.00
	Associate senior professional	8	50.00	6	50.00	6	50.00
Workplace	Water supply authorities	3	18.75	3	25.00	3	25.00
	Academic institutions	4	25.00	3	25.00	3	25.00
	Centre for Disaster Management	1	6.25	1	8.33	1	8.33
	Regulatory authority	1	6.25	1	8.33	1	8.33
	NEMC	2	12.50	1	8.33	1	8.33
	Consultancy and contractors	2	12.50	1	8.33	1	8.33
	LGA	1	6.250	1	8.33	1	8.33
	Ministry of water	2	12.50	1	8.33	1	8.33
Disaster experience	Yes	11	68.75	9	75.00	9	75.00
	No experience	5	31.25	3	25.00	3	25.00

**Total**		16	100.00	12	100.00	12	100.00

LGA, Local Government Authority; NEMC, National Environmental Management Council.

#### First-round assessment

Of the 16 respondents in this round, eight participated for the first time. Results show that 75% of the experts provided comments strongly reflecting on the tool in the current study. Experts’ opinions that emerged from this round were analysed and summarised as for latter addition, revision or integration in the study. Economic dimension was defined as the ability of economic entities, such as individuals, households, societies, water supply authorities and firms, to use their economic resources to quickly recover or adjust to the loss of WSSs because of flooding impacts. The dimension encompassed three principles (see Table 7). There were no significant changes concerning the indicators – a few specific comments entailed minor changes or improvement to the descriptions. For instance, ‘system investment proportionality’ description changed – the word equality was replaced by proportionality; thus, the description was improved to the proportionality of investment from the system production and transmission to system distribution such as to ensure uniformity in services and reduce losses.

**TABLE 7:** Improved principles for economic dimension resilience.

Principles	Description
Structure	The composition and patterns of various components of the economy such as trade, income, employment, etc., ranging from water users to the organisations that run the WSSs
Security/static	The ability of an entity or system (household, society or organisation) to maintain function by making the best use of available resources. It is essentially concerned with the efficient allocation of resources, and it principally involves users (customers)
Dynamism	The efficient use of resources over time for investment in repair and reconstruction focusing on the speed of recovery of water supply from the impacts of flooding

WSS, water supply systems.

#### Second-round assessment

The tool improved from first-round assessment comprised three principles and 12 indicators. In the second round, experts ranked the dimensions and principles and rated the indicators based on their importance. Dynamism was ranked the most important principle with higher frequency (five times) than any other principle. It was also associated with the lowest mean (1.75) and median (2.00) than others. Other principles had relatively high mean and median; besides, the structure principle had a higher mean (2.25) and median (2.50) than security/static (2.08 and 2.00) for mean and median, respectively. As such, the ranking order of the importance of principles started with 1 for dynamism, 2 for security and 3 for structure.

**Rating of indicators:** The six attributes applied as useful guidance in rating the importance of the indicators and their potential for inclusion in the study. Despite all indicators passing the relevance attribute, none of the indicators passed all six attributes. The majority (75%) were lowly rated for at least three out of the six attributes. ‘Stability of prices and incomes’, and ‘Business cooperation (intra and inter)’ were the lowest rated indicators – they underrated five out of the six attributes. Only ‘public–private-partnership’, ‘system investment proportionality’ and ‘cost recovery’ indicators were affected by one of the six attributes. Of all attributes, data availability was the major concern affecting most – 83.3% – indicators.

Results indicate that nine indicators equivalent to 75% were excluded from the study, prompting for the exclusion of two principles – structure and security. Excluded indicators also encompassed relatively low mean ratings and higher standard deviations than others. Such results suggest that the group opinions for their inclusion were low and the importance rating had low convergence compared with others. The lowest rated indicators include ‘business cooperation (intra and inter)’, ‘individual and community savings’ and ‘stability of prices and incomes’, as less than 50% of the experts thought they were important.

Only three indicators were included in this study at this stage – ‘public–private-partnership’, ‘cost recovery’ and ‘system investment proportionality’. The importance of all three indicators was supported by 75% of the experts. Besides, ‘system investment proportionality’ was regarded the most important indicator because of higher mean rating (3.7500) and lower standard deviation than others. The second important was ‘public–private-partnership’, whereas ‘cost recovery’ was the least important indicator.

#### Third-round assessment

The improved tool included one principle and three indicators. During the third round, the majority (83.3%) of the experts did not revise their opinions because they were satisfied with the components of the tool, which had emerged from the second round. Some experts provided the following responses – ‘I have gone through the indicators, and I find at the stage you have reached all suffice the assessment; that said I have no any additional input to it’, ‘very sorry for the late response; yes please, kindly proceed with further steps, I don’t have different opinions’ and ‘I think there are no changes, you can proceed’.

On the contrary, 16.7% of the experts reconsidered their voting for re-ranking the principles and rerating the indicators. Statistical results comparing the second-round and third-round principles’ ratings show a *p*-value of 0.4875, suggesting that there was no significant difference between the rounds. Similarly, statistical analysis comparing the importance rating of the indicators between the second round and the third round shows *p*-values ranging from 0.3949 to 0.4862, indicating no significant difference between the rounds. The results suggest that there was enough consensus, and experts were satisfied with the indicators – thus, all three sufficed for inclusion in this study. The mean value ranged from 3.667 to 3.750 and standard deviations from 0.754 to 0.888 – the overall convergence of the importance ratings can be considered acceptable. The Kappa values for the components of the tool ranged from 0.852 to 1.000 (mean = 0.9507, median 1.000). These values suggest that there was a substantial agreement between the two rounds – the final tool is presented in Table 8.

**TABLE 8:** Tools for measuring the economic resilience for water supply systems in Tanzania.

Principle (MS, MeS)	Indicators		*p*-value	Kappa score	Rank
	Indicator (MS, SD)	Description			
4.1 Dynamism *(1.75, 2.00) (0.4875, 1.000)*	4.1.1 System investment proportionality *(3.750, 0.754)*	Proportionality of investment for the system from production and transmission system to distribution network such as to ensure uniformity in services and reduce losses	0.4862	1.000	1
	4.1.2 Public–private partnership (PPP) *(3.667, 0.888)*	The partnership between the water supply authority and private sector, including private water services companies, in the delivery of water service during flooding	0.3949	0.852	2†
	4.1.3 Cost recovery *(3.667, 0.888)*	Recovering the costs of any given expense regarding operation and maintenance of the WSS through the billing	0.4862	1.000	2†

MS, mean score; MeS, median score; SD, standard deviation; WSS, water supply system.

† , they are equal in terms of their importance.

## Discussion

The study adopted a three-stage approach – literature review, pre-assessment and Delphi survey – to develop a tool suitable for assessing the economic dimension resilience for WSSs in Tanzania. A tool comprising of nine indicators was proposed from the literature. The tool first derived from the literature included features that could assess the internal and external economic resilience of the WSSs at the microeconomic and mesoeconomic level. The tool, later, passed through a pre-assessment exercise that involved 10 water supply experts. Expert’s opinions were analysed, and the results were used to improve the tool. The improved tool comprising of three principles – structure, static and dynamism – and 12 indicators was further subjected to a three-round Delphi exercise. The experts who provided feedback were between 12 and 16, which are considered minimally sufficient participants (Hsu & Sandford 2007) for a successful Delphi exercise. The rate of response increased from 72.7% in the first round to 100% in the final round, which is in line with Gargon et al. (2019) who suggest that small size panels are likely to have significantly better response rates. The rates were enhanced by participants’ contact at the beginning of each round and regular reminders to provide feedback.

Results show that there was no substantial changes or modifications that emerged from the first round – the only comments were associated with two indicators, ‘expenditure on water services’ and ‘system investment proportionality’. Experts suggested that the description of the expenditure on water services should consider other unpaid resources, such as volunteering works. For the systems investment proportionality, they suggested changes in the description from equal investment to proportional investment. Most of the indicators that were excluded during the second round were associated with data availability, affordability and reliability. Indicators that were under-rated for those three attributes could not qualify for inclusion in this study, and the majority were excluded. The three attributes express concerns in the country as in most other developing countries. For instance, Nobert (2016), UN-WATER (2013), URT (2008) and World Bank (2018), concurred with the current findings that the lack of consistent and accurate data is a typical limitation in the country. The findings suggest that the three attributes are principle factors when choosing indicators in the country and other developing countries.

Most experts were satisfied with the tool that involved one principle and three indicators after the second round, such that they did not revise their voting in the subsequent round. Consensus building in two iterations is no strange as other studies, such as Suwaratchai et al. (2011), were able to obtain consensus during the second round. Some experts reconsidered their voting during the third round – statistical results indicated that the importance ratings for indicators between the second and third rounds had no significant difference. The mean scores and standard deviation values suggest that there were better group opinions and that the overall convergence of the importance ratings can be considered acceptable. Moreover, Kappa statistic values for all components of the tool ranged from 0.852 to 1.000 – thus, according to Zhong et al. (2015), the values suggest that there was a substantial agreement between the two rounds and that the consensus had been reached.

**FIGURE 2:**
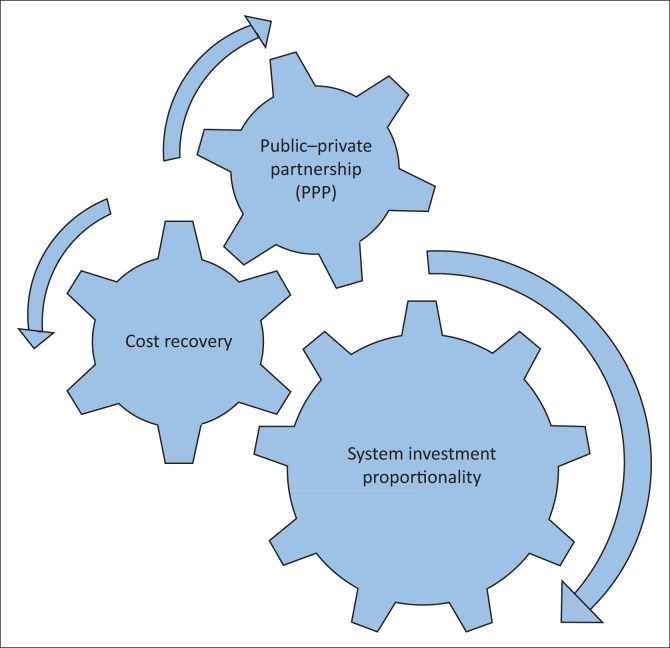
New model of economic resilience for water supply systems in Tanzania.

The tool underwent significant changes at the end of the Delphi exercise, excluded indicators (75%) prompted for the automatic omission of two principles – structure and security. That said, dynamism principle, which is associated with how fast the system could recover from flood hazards and return to its normal condition, was in favour of the experts and remained the only principle for the tool. Besides, only three indicators – system investment proportionality, cost recovery and PPP – were included in the dynamism principle of the tool. The first two indicators assess the internal economic resilience – for instance, cost recovery indicates the capacity of the organisations to generate own financial resource through billing leading to availability of contingency funds, which could facilitate rapid recovery of services aftermath. The system investment proportionality depicts the efficient use of the financial resources in such a proportion that could not affect the system functionality. The last indicator – public–private partnership assesses the external economic resilience, it describes the adaptive capacity that could be enhanced by assistances from other partners during flooding. In all cases, system investment proportionality is the most important indicator followed by both public–private partnership and cost recovery indicators. The findings suggest that the economic function of the water supply at the organisations level is vital for building WSSs’ economic resilience against flood hazards. The new model of economic resilience for WSSs in Tanzania is presented in Figure 2.

The current study was conducted through a review of various international economic resilience attributes from different frameworks and made relevant to Tanzania through experts’ involvement. Thus, the study entails a more needed beginning for broad agreement about the components of WSSs’ economic resilience against flood hazards in Tanzania. To date, studies on the water supply field in Tanzania have been focused on water management, climate change studies and floods risk analysis with limited focus on economic resilience. Thus, the current tool is useful in evaluating the economic dimension resilience for WSSs in the country. The tool is supported by a management approach of enabling water supply organisations to provide water supply services sustainably when faced with flood hazards.

The tool can be used by water supply professionals and managers to assess the economic resilience for WSSs using their internal data. The tool can also be used to identify priority activities that can assist in enhancing resilience and consequently address future flood hazards. Finally, the tools can be applied in other developing countries, as the agreed measures were devised from general concepts of the economic resilience from the literature.

## Conclusion

The study developed a tool with a principle and key indicators of the economic dimension resilience for WSSs in Tanzania. It provides a potential beginning for broad agreement regarding the key components of the economic resilience for WSSs in Tanzania. The economic components are the principle requirements enabling WSSAs to have economic capacity and collaboration in enhancing technical, environmental, organisational and social resilience for the WSSs. For instance, cost recovery ensures availability of contingency funding that can support the resilience building activities. The tool can be used for evaluating and informing the priority practices that can assist water supply organisations in Tanzania and other developing countries to address future impacts of flood hazards. Like other qualitative tools, the assessment process can be affected by subjectivity, but this can be reduced using a group of experts during the assessment process. Data availability and quality would be another limitation; however, with proper data management systems, WSSAs can ensure a smooth assessment process.
